# Recent Advancements in Cell-Based Therapies in Melanoma

**DOI:** 10.3390/ijms25189848

**Published:** 2024-09-12

**Authors:** George Nassief, Angela Anaeme, Karen Moussa, Abdallah N. Mansour, George Ansstas

**Affiliations:** 1 Division of Medical Oncology, Department of Medicine, Washington University in Saint Louis, Saint Louis, MO 63110, USA; 2 UMKC School of Medicine, University of Missouri Kansas City, Kansas City, MO 64108, USA; 3 Department of Medicine, National and Kapodistrian University of Athens, 15772 Athens, Greece

**Keywords:** tumor-infiltrating lymphocytes, melanoma, bi-specific T-cell engagers, CAR T therapy, cell-based therapy

## Abstract

Malignant melanoma outcomes have drastically changed in recent years due to the introduction of immune checkpoint inhibitors (ICIs). However, many patients still experience intolerable side effects, therapy resistance, and disease progression on ICI therapy. Therefore, there remains a need for novel therapeutics that address this gap in treatment options. Cell-based therapies have gained wide attention as a therapeutic option that could address this gap in treatment options for advanced melanoma. These therapies work by extracting certain cell types produced in the human body such as T-cells, modifying them based on a specific target, and transfusing them back into the patient. In the realm of cancer therapy, cell-based therapies utilize immune cells to target tumor cells while sparing healthy cells. Recently, the Food and Drug Administration (FDA) has approved the usage of lifileucel, a tumor-infiltrating lymphocyte (TIL) therapy, in advanced melanoma. This came following recent results from the C-144-01 study (NCT02360579), which demonstrated the efficacy and safety of TILs in metastatic melanoma patients who otherwise failed on standard ICI/targeted therapy. Thus, the results of this trial as well as the recent FDA approval have proven the viability of utilizing cell-based therapies to fill the gap in treatment options for patients with advanced melanoma. This review aims to provide a comprehensive overview of major cell-based therapies that have been utilized in melanoma by delineating results of the most recent multi-center phase II/ III clinical trials that evaluate the efficacy and safety of major cell-based therapies in melanoma. Additionally, we provide a summary of current limitations in each cell-based therapeutic option as well as a future direction of how to further extrapolate these cell-based therapies in advanced melanoma.

## 1. Introduction

Malignant melanoma patient outcomes have changed drastically with the emergence of immune checkpoint inhibitors (ICI) and BRAF-targeted therapies and the lethality of advanced disease has decreased significantly in the past 10 years [1,2]. In 2011, Ipilimumab was first approved to treat advanced melanoma patients by blocking the action of cytotoxic T-lymphocyte-associated antigen 4 (CTLA-4) [3]. Following this, pembrolizumab and nivolumab, programmed death receptor 1 (PD-1) inhibitors, were approved and were recognized as standard of care for advanced melanoma patients [4]. The landmark CheckMate-067 trial showed that the combination of ipilimumab–nivolumab in advanced melanoma patients has a 48% overall 7.5-year survival [5]. However, the problem of primary and acquired resistance to ICI therapy still interferes with patient response to such treatments [6,7,8]. Moreover, a significant number of patients experience intolerable side effects and disease progression during ICI treatment [9,10,11]. Therefore, there remains a call for new therapeutics for a significant number of melanoma patients who may not benefit from ICI therapy.

Cell therapies have been extensively researched in recent years as a therapeutic option for advanced melanoma patients. Cell-based therapies work by extracting certain cell types from the human body, modifying them toward a target, and transfusing them back into the patient. They are often utilized in cancer therapy as they can enhance the immune system’s ability to fight malignancies. Importantly, advancements in genetic engineering in cell therapies, such as engineered T-cell receptor (TCR) therapy, allow for T-cells to only target tumor cells while sparing healthy cells. A major milestone in cell-based therapies was reached upon the recent Food and Drug Administration (FDA) approval of tumor-infiltrating lymphocytes (TILs) in advanced melanoma. Here, we aim to provide a review of recent advancements in major cell-based therapy treatments in melanoma by presenting the most recent multicenter phase II/phase III clinical trial results that highlight the efficacy and safety of this therapeutic option. Furthermore, we aim to discuss current limitations in cell-based therapies as well as provide possible future directions as to how to implement this treatment option in the therapeutic arm against melanoma.

## 2. TILS Therapy in Melanoma

TILs are a novel treatment option that relays promise in melanoma therapy [12,13]. Lymphocytes play an important role in tumor dissemination as they can recognize abnormal cells and penetrate tumors [14,15,16]. This has made this treatment option ideal for cancer therapy. The process of TIL therapy starts with tumor resection and isolation. Then, ex vivo expansion of the TILs and activation within the resected tumor takes place. These expanded and activated TILS are then transfused back into the patient following lympho-depletion therapy with the hope that they are primed and activated against the tumor they reside in [17,18]. Following infusion, interleukin 2 (IL-2) is administered to enhance the in vivo expansion of the administered T-cells [19,20].

Rosenberg et al. were the first to demonstrate TIL therapy efficacy in melanoma. In their study, 20 metastatic melanoma patients received adoptive TIL therapy along with IL-2. Of the patients who did not receive prior IL-2 treatment, 9 out of the 15 patients demonstrated objective cancer regression in sites such as the lung, bone, and skin, which lasted for 2 to more than 13 months. Toxic effects following IL-2 treatment were observed, although they were reversible [18]. Thus, this study demonstrated the efficacy of TIL therapy in metastatic melanoma and paved the way for future research to investigate this treatment option in other solid tumor malignancies.

Recently, the FDA approved the first cell-based therapy in advanced melanoma, lifileucel, which is also commercially known as Amtagvi. Lifileucel, a TIL therapy, was approved for patients who previously progressed on standard immunotherapy/targeted therapies. This is a milestone in advanced melanoma treatment given that there are no approved treatment options following standard-of-care immunotherapy/targeted therapy. Thus, this therapeutic approach could address the gap in advanced melanoma treatment options available for patients who otherwise fail standard therapy.

FDA approval came following recent results presented from the C-144-01 study, a phase II clinical trial evaluating the efficacy and safety of lifileucel in patients with metastatic melanoma who failed on standard ICI/targeted therapies (NCT02360579). In the study, the median number of TIL cells infused was 21.1 × 10^9^, with a range of 1.2 × 10^9^ to 99.5 × 10^9^. The study had previously demonstrated that lifileucel, a one-time autologous TIL therapy, had a durable response and demonstrated an objective response rate (ORR) of 31.4% in heavily pre-treated patients with ICI therapy [21]. Recently, the study presented its four-year analysis of the usage of lifileucel in pre-treated melanoma patients. In the 153 assessed patients and with a median follow-up of 48.1 months, the study observed a 1-, 2-, 3-, and 4-year overall survival (OS) rate of 54.0%, 33.9%, 28.4%, and 21.9%, respectively. When broken down by different patterns of responses, the 4-year OS rate ranged from 37.2% to 68.2%, with more deepened responses having higher OS rates. The study also reported no new safety concerns other than the known adverse events related to TIL therapy and IL-2 [22]. These results provided great promise as they demonstrated a durable clinical response to TIL therapy in patients who otherwise failed standard-of-care therapy and highlighted the efficacy of lifileucel as a therapeutic option for advanced melanoma patients. Thus, this paved the way for the approval of TIL therapy in melanoma and warranted investigation into how this treatment option could fit into the therapeutic approach against melanoma.

Another phase 3 clinical trial recently presented data evaluating the safety and efficacy of TIL therapy (treatment group) against ipilimumab (control group), the traditional second-line treatment for melanoma patients (NCT02278887). A minimum of 5 × 10^9^ TILS cells were at least infused. In the trial, TIL therapy demonstrated a higher progression-free survival (PFS) in advanced melanoma patients at 7.2 months compared to 3.1 months found in the ipilimumab control group. In addition, patients treated with TIL therapy had an objective response rate (ORR) of 49% compared to 21% for the ipilimumab treatment group, with a hazard ratio (HR) for progression or death of 0.50 between the treatment and control group (*p* < 0.001). Furthermore, the TILs treatment group had a median overall survival of 25.8 months compared to 18.9 in the ipilimumab group. In the trial, they discovered a higher frequency of treatment-related events (TRAEs) in the TILs treatment group with all patients experiencing ≥ grade 3 TRAEs compared to ipilimumab with only 57% of patients experiencing ≥ grade 3. The higher rate of TRAEs was mainly due to lymphodepletion chemotherapy, IL-2, or the synergic effect of both. However, a higher frequency of global health-related quality-of-life scores was indicated in the TILs group based on patients’ responses [23]. Therefore, the ORR along with the PFS relays TILs as a viable first- or second-line treatment option for patients with metastatic melanoma. Furthermore, many clinical trials evaluating TIL therapy as a monotherapy or in combination with other therapeutic options are ongoing to evaluate how this treatment option could fit into the treatment algorithm against melanoma (Table 1).

However, the treatment comes with several challenges and more research is warranted to explore ways to improve the therapeutic delivery of TILs. Challenges such as the high cost of manufacturing and the treatment being mainly centralized in centers of expertise preclude many patient populations in rural areas from benefiting from this treatment [24]. Delivering elements of TIL therapy in an outpatient setting may become available in the future with experience and thus reduce the cost associated with the treatment. However, such a program requires much coordination and a multifaceted approach to treatment delivery that requires further scrutiny [19]. Another challenge present with TIL therapy is the long waiting time needed to harvest and expand the T-cells. A solution to this would be an earlier harvesting of TILs prior to treatment administration [25]. However, a logistical approach to this remains unclear as of now.

Finally, an important consideration that requires future investigation for this new therapeutic approach is optimal patient selection for TIL therapy. The study investigating lifileucel pointed out that a shorter anti-PD-1 therapeutic course could maximize the TILs therapy response period [26]. Lifileucel was also reported to be less effective in patients with large tumor burdens- those with a higher cancer spread in their body [27]. Therefore, more research is required to uncover potential biomarkers for TIL therapy patient selection as in the case of ICI therapy to optimize the outcomes of this treatment approach.

Overall, TIL therapy has demonstrated great promise in melanoma treatment as demonstrated by the recent FDA approval of lifileucel. Beyond melanoma, TIL therapy has shown promise in anti-PD-1-resistant metastatic lung cancer [28] and tumor eradication in cases of advanced colon and breast cancer [29,30]. Thus, the treatment is widening therapeutic options for advanced cancer patients and many initiatives are being undertaken to mediate the challenges present with TIL therapy [27]. 

## 3. Modified Cell Therapies in Melanoma

Similar to TILs, genetically modified cellular therapies take advantage of the intrinsic ability of the immune system to recognize and target specific antigens and foreign substances. Cellular therapies used in cancer treatment include both autologous and allogeneic immune cells, with the former originating from the patient’s own immune cells and the latter originating from cells harvested from other individuals [31]. The efficacy of genetically modified cellular therapies in cancer treatment was first demonstrated in 2006 by Kershaw et al., who employed genetically modified T-cells to treat ovarian cancer [32]. The promising results from this phase I clinical trial provided the basis for future studies to further investigate the use of modified cell therapies in other cancer subtypes, including neuroblastoma, blood cancers, and, more recently, melanoma [33,34,35]. As understanding of the unique immunogenic properties of each type of immune cell has developed, so too has the ability to develop cancer therapies that target cellular processes more specifically and directly. Below is a discussion of the most prominent genetically modified cell therapies in the realm of melanoma treatment.

### 3.1. TCR-Transduced T-Cell Therapy in Melanoma

One of the primary types of modified cell therapies is TCR-transduced T-cell therapy. TCR therapy leverages the ability of transmembrane receptors on T lymphocytes to recognize antigens presented on major histocompatibility complexes (MHCs) of antigen-presenting cells as foreign bodies and initiate an immune response [36]. In the case of melanoma treatment, the antigens that TCR-transduced T-cells are primed to recognize are tumor-specific antigens. The process of employing TCR therapy begins with extensive patient screening, including HLA typing. Tumors are then biopsied to analyze the tissue for tumor-specific antigens to target. Subsequently, patients undergo leukapheresis to isolate T-cells, which are then pre-activated with anti-CD28 and anti-CD3 antibodies. Then, TCRs specific to the tumor antigen to be targeted are isolated from healthy donors and transferred into the patient’s isolated T-cells using a lentivirus vector. Following the expansion of these genetically modified T-cells, TCR therapy is infused into the patient, followed by a low dose of IL-2 [37].

The first clinical trial to demonstrate the utility of TCR-transduced T-cells in melanoma was conducted by Johnson et al. in 2006 (NCI-07-C-0174/NCI-07-C-0175). This study utilized transgenic mouse models to generate TCRs specific to melanoma antigens, specifically MART-1 (Melan-A). Upon infusion of genetically modified T lymphocytes with these melanoma-specific TCRs into 36 patients with metastatic melanoma, favorable outcomes were achieved, with high persistence of genetically modified lymphocytes in patient serum one month after treatment. Additionally, 30% of patients who received human TCR and 19% who received mouse TCR demonstrated objective cancer regression. Notably, however, negative side effects included destruction of normal melanocytes in the skin, eye, and ear; uveitis; and hearing loss [38]. This trial laid the foundations for further exploration of both the benefits and pitfalls of TCR therapy in melanoma.

Subsequent trials built off these findings and sought to further corroborate the efficacy of MART-1-specific TCR-transduced T-cells in melanoma treatment. A phase II trial investigating the safety and efficacy of MART-1 TCR transgenic lymphocytes and MART-1 peptide-pulsed dendritic cell (DC) vaccination double therapy in patients with metastatic melanoma was conducted (NCT00910650). This same trial also compared freshly manufactured TCR-transduced T-cells with cryopreserved lymphocytes. The trial assessed a dosage of up to 1 × 10^9^ cryopreserved TCR transgenic lymphocytes in the first patient cohort and increased it to up to 1 × 10^10^ cryopreserved TCR transgenic lymphocytes due to suboptimal antitumor activity. A short one-week manufacturing protocol of genetically modified T-cells was conducted, addressing the long wait time of this treatment modality. The trial found 69% (n = 14) of patients demonstrating tumor regression, with rapid in vivo expansion occurring two weeks after infusion. Furthermore, the trial found higher persistence of transduced T-cells after freshly manufactured T-cells were infused, compared to cryopreserved T-cells. There was no robust evidence of the utility of DC vaccines in enhancing in vivo expansion [39]. Nonetheless, the trial illustrated the feasibility of a shorter course manufacturing protocol of TCR therapy, as well as the utility of MART-1-specific TCR therapy in melanoma treatment.

Establishing the utility of MART-1-specific TCR transduced T lymphocytes in melanoma was critical to the validation and acceptance of TCR therapy as a treatment modality, as up to 95% of melanoma tumors express MART-1 [40,41]. However, other common melanoma antigens have also been investigated as targets for TCR therapy. A phase II trial evaluating the safety and efficacy of TCR therapy in metastatic melanoma looked at NY-ESO-1 tumor antigen as a potential target, which is expressed in approximately 25% of melanoma patients (NCT00670748). Patients with refractory metastatic melanoma and synovial cell sarcoma with NY-ESO-1-positive tumors received antigen-specific TCR-transduced T-cells, along with IL-2 following chemotherapy. The trial assessed a median dosage of 5 × 10^10^ (range of 1.6 × 10^9^ to 130 × 10^9^) TCR transduced T-cells. Of the 11 treated melanoma patients, 5 (45%) had objective clinical responses, and 2 (18%) had complete tumor regression that persisted after one year. In contrast to clinical trial findings of MART-1-specific TCR therapy, NY-ESO-1-specific TCR-transduced lymphocytes had no off-target toxicities in melanoma patients due to the preferential expression of NY-ESO-1 on tumor cells and its absence in normal tissues [42]. These findings indicate a substantial benefit of NY-ESO-1 and other highly specific tumor antigens as targets of TCR therapy by eradicating the concern of adverse systemic reactions. Overall, this trial supports the efficacy of NY-ESO-1 TCR therapy and opened the door for further exploration of other viable tumor antigens as TCR therapy targets.

In addition to MART-1 and NY-ESO-1, melanoma-associated antigen (MAGE) has been a target of much interest in TCR therapy. The family of MAGE genes has been described as an ideal target for TCR transduced cell therapy, as it is highly expressed in several different tumor types, and expression in healthy native cells is restricted to the testis and placenta [43]. However, clinical trials seeking to demonstrate the efficacy of MAGE TCR therapy in cancer treatment have demonstrated notable toxicities caused by TCR crosslinking, halting its uptake as a mainstay treatment in melanoma and other cancers [44]. In fact, a large phase III clinical trial seeking to investigate the treatment of metastatic melanoma with GSK 2132231A, an anti-MAGE-A3 TCR-transduced T-cell therapy, was prematurely terminated due to prominent negative side effects and lack of evidence of efficacy (NCT00796445) [45]. Additional clinical trials investigating anti-MAGE TCR therapy in melanoma have yielded similar results. Clinical trial results published by Morgan et al. found that five out of nine enrolled patients demonstrated clinical regression of their melanoma; however, three patients had notable mental status changes, and two patients entered a coma before dying just two days after treatment infusions (NCT01273181) [46]. Thus, despite characteristics that once suggested the potential utility of MAGE antigens as a target for future cancer therapies, there has been limited evidence of the efficacy and safety of anti-MAGE TCR-transduced T-cells. Further investigation of the unique features of anti-MAGE TCR therapy that may be driving these toxicities is necessary.

Currently, there are no TCR-transduced T-cell therapies that have been approved by the FDA for advanced or metastatic melanoma. Despite demonstrating promise as a potential mainstay of melanoma treatment, there are still several limitations to TCR-transduced T-cell therapy that must be considered. As mentioned previously, on- and off-target toxicities pose a threat to the suitability and safety of TCR therapy among recipients. Furthermore, a challenge in implementing TCR therapy is the specificity of the antigens to be targeted. Development of the transduced T-cells requires identification of the tumor antigens for targeting; thus, it is only possible to target antigens that are already well defined, limiting the applicability of TCR therapy to unknown antigens or tumors with heterogeneous makeup. Additionally, because the antigen expression profiles of melanoma can differ between patients, it is possible that a highly specific TCR therapy may not consistently yield favorable results between patient populations, leading to inconsistent efficacy [31]. Lastly, the manufacturing process of TCR-transduced T-cells is very time- and resource-intensive, and these logistical challenges must continue to be addressed as this modality is explored in the future [37,47].

Another important consideration that limits the clinical applicability of TCR-transduced T-cell therapies is HLA restriction. Patients given TCR therapy must express both the target antigen as well as the antigen-restricting HLA allele [48]. Therefore, this can be very limiting to patients across different ethnic groups who express the most commonly utilized HLA allele to a lesser frequency. For instance, HLA-A*02:01, the most common HLA allele utilized in TCR-transduced T-cell therapies, is more frequently expressed in Europeans (27% allele frequency) and is less prevalent in African Americans (11.9%) and people of Asian or Pacific Island ancestry (6.5%) [49,50]. Therefore, this is a significant shortcoming of this therapeutic approach as it rules out patients from different ethnic backgrounds from benefiting from this treatment option. Thus, more research is needed to overcome this shortcoming by exploring HLA subtypes that are more prevalently expressed in patients from ethnic backgrounds who otherwise do not express the HLA-A*02:01 subtype as prevalently.

### 3.2. CAR T-Cell Therapy in Melanoma

Another modified cell therapy that has shown great promise in melanoma treatment is chimeric antigen receptor (CAR) T-cell therapy. In contrast to TCR-transduced T-cell therapy, which uses TCRs that are naturally occurring on lymphocytes, CAR T-cell therapy utilizes synthetic, genetically engineered receptors that recognize surface antigens of tumor cells [51]. Since CAR therapy recognizes natively folded proteins at the cell surface rather than HLA antigens, CAR therapy presents a major therapeutic advantage over TCR therapy since it can overcome the HLA restriction, which limits many patients from benefiting from TCR therapy [48]. Manufacturing CAR T-cells begins with the isolation of patient T-cells from the blood via leukapheresis. Then, these T-cells are modified in the lab by inserting a gene encoding a genetically engineered CAR—synthesized to recognize a specific antigen—into the patient’s T-cells. The lymphocytes are then left to proliferate and grow in the lab before being reinfused back into the patient. Once the patient has received the infusion, the genetically modified CAR T-cells recognize the tumor-specific antigens and attack the targeted cancer cells [52]. Additionally, upon antigen recognition and binding, the CAR T-cells release cytotoxic agents such as granzymes and perforin, further stimulating cancer cell death [53].

Since the approval of CAR T-cell therapies for blood cancers by the FDA in 2017, CAR T-cell therapy has gradually gained traction as a mainstream cancer treatment [54]. However, there is less robust evidence for the utility of CAR T-cells in solid tumors, especially melanoma. There is a paucity of completed clinical trials that investigate the use of CAR T-cells in melanoma: as of now, there are only two completed clinical trials on the matter, and only one has released its results. This phase I/II clinical trial utilized CAR T-cells that were specific to Vascular Endothelial Growth Factor Receptor 2 (VEGFR2) and studied a cohort of 24 patients to determine the safety and efficacy of this treatment (NCT01218867). Ultimately, the trial was terminated, as no objective responses were observed and numerous grade 3/4 adverse events were recorded, including nausea, vomiting, hypoxia, and elevated levels of aspartate transaminase, alanine transaminase, and bilirubin [55,56]. These findings suggest the need for further understanding of the characteristics of this treatment regimen that may have yielded unfavorable results, as well as a pivot to other potential targets of CAR T-cell therapy for melanoma.

Despite these discouraging trial results, active clinical trials are underway to identify potentially more appropriate melanoma antigens that CAR T-cells can target, as well as simultaneous therapies that can improve the success of CAR T-cell therapies. Some targets currently under investigation in clinical trials include GD2, cMet, hCD70, CD20, and several others [57,58]. Indeed, the only other completed clinical trial evaluating the efficacy of CAR T-cell therapies in melanoma focused on GD2 as a target of CAR T-cell therapy in solid tumors (NCT02107963). Although these results were not posted, the elucidation of novel targets of CAR T-cell therapy is a pertinent topic of interest in cancer therapeutics today. Included in the table below are the current ongoing clinical trials examining the use of CAR T-cell therapy in melanoma.

There are several potential explanations for the scarcity of clinical trials supporting the use of CAR T-cells in melanoma, as well as solid tumors at large. Many logistical challenges exist in the implementation of this therapy modality. Not only does the process of manufacturing each CAR T-cell require extensive time and resources, but the cost also makes this treatment inaccessible for many patients, with some therapies costing over USD 450,000 [57]. This financial burden—along with the lack of knowledge and health literacy surrounding this relatively novel and complex treatment—poses a barrier to several patients who might be candidates for this therapeutic option.

There are also several side effects associated with CAR T-cell therapy that may raise concern for potential recipients, including fevers, hypotension, severe confusion, seizures, and impaired speech [57]. However, the most serious and most common side effect of CAR T-cell therapy is cytokine release syndrome (CRS). This potentially fatal complication occurs when the infused CAR T-cells induce a rapid and massive release of cytokines, which stimulate the body’s immune response and result in dangerously high fevers, severe hypotension, hypoxia, and capillary leak. CRS most often occurs in patients with more extensive and advanced cancers; however, this complication can be managed with steroids, and other forms of supportive care are currently being investigated [59]. Nonetheless, CRS and other adverse side effects complicate the widespread use of CAR T-cell therapy in melanoma.

Other important considerations in the implementation of CAR T-cell therapy in melanoma include tumor heterogeneity, antigen loss, immunosuppressive tumor microenvironment, and insufficient infiltration or penetration of T-cells into the tumor [58]. Such challenges may be responsible for the differences in efficacy and response rate between CAR T-cell therapy and other treatment modalities. For instance, some studies have found that, although CAR T-cell therapy has comparable response rates and overall survival to other standard treatment options, patients receiving CAR T-cells may have higher rates of relapse and disease progression [60]. However, there are efforts underway to address these challenges. For instance, CAR T-cells that are resistant to immunosuppressive molecules in resistant tumor microenvironments, express enzymes that can degrade extracellular matrix, encode genes that induce cell death, or secrete cytokines to overcome the complex microenvironment are being developed to address some of these challenges [58]. Thus, despite the progress that must be made in CAR T-cell therapy options for melanoma treatment, there are grounds for hope on the horizon that further progress will be made on this front, and future clinical trial results will help delineate how CAR T-cell could be further extrapolated in melanoma therapy (Table 2).

### 3.3. NK Cell Therapy in Melanoma

Another adoptive cell-based therapy that has been explored in melanoma in recent years is natural killer (NK) cell therapy. NK cell therapy presents with major therapeutic advantages compared to other modified cell therapies. For instance, unlike other cell-based therapies, NK cell therapy does not require TCR-HLA interactions and is unimpacted by tumor escape through the MHC pathway [61,62]. The main mechanism of action by which NK cell therapy eradicates tumor cells is through the release of lytic granules, such as perforins and granzymes, in target cells [63]. Additionally, NK cell therapy works through other mechanisms of action, such as antibody-dependent cellular toxicity and death receptor-mediated pathways [62,63]. Furthermore, NK cell isolation from different sources, such as umbilical cord blood, peripheral blood mononuclear cells, and differentiated induced pluripotent stem cells, has been investigated to optimize this therapeutic approach [64].

NK cell therapy has demonstrated multiple potential clinical advantages that require further extrapolation in melanoma therapy and translation in large multi-center clinical trials. However, the treatment comes with many challenges that require further research. For instance, difficulties in ex vivo expansion of the treatment, insufficient infiltration in solid tumors, as well as how an immunosuppressive tumor microenvironment could impact the treatment’s clinical efficacy are all limitations of this adoptive cell therapy [65]. However, research into how different sources of NK cells and different expansion methods for the therapy are underway to optimize the clinical efficacy of the drug and overcome the challenges present with this therapeutic approach [65]. Importantly, many clinical trials are currently underway to evaluate the efficacy and safety of this therapeutic approach as well as to help in understanding how this treatment option could fit into the clinical approach against melanoma (Table 2).

## 4. Bi-Specific T-Cell Engagers

Bispecific T-cell engagers (BiTEs) are a novel antibody therapy that has been used in the treatment of hematologic malignancies [66]. This treatment choice has shown promising therapeutic effects in treating metastatic melanoma, including cutaneous melanoma resistant to PD-1 and CTLA-4 inhibitors [67,68] and metastatic uveal melanoma [69]. Here, we will discuss the mechanism of action of BiTE therapy as well as highlight current clinical trials that investigate the usage of this therapeutic choice in melanoma.

Mechanistically, BiTEs are designed to redirect the patient’s T-cells to specifically target tumor cells, thereby enhancing the body’s immune response against the tumor. The therapy works by binding to both T-cells and tumor cells, having two single-chain fragment variables (scFv): one that attaches to a protein on the surface of T-cells, usually CD3, and another that targets a specific antigen found on the surface of tumor cells, also known as a tumor-associated antigen (TAA). By bringing these two cell types into close proximity, BiTEs help activate T-cells and direct them to attack malignant cells, leading to malignant cell lysis [70,71]. In melanoma, Gp100 is one of the glycoproteins expressed on the surface of the tumor cells. Gp100 serves as the TAA in BiTEs. Upon binding to CD3 on T-cells, the BiTE forms an immunological synapse between the T-cell and the melanoma cell. This synapse enables the focused release of perforin and granzymes from the T-cell, leading to apoptosis of the melanoma cell. Additionally, the engagement of CD3 leading to T-cell activation promotes the release of cytotoxic granules and cytokines, which improves the local immune response, recruiting and activating more T-cells [72]. Importantly, unlike many other T-cell-based therapies, BiTE-mediated tumor cell death is independent of both HLA expression and TCR and can occur in the absence of costimulatory signals [70,73,74].

Uveal melanoma, a melanoma subtype that impacts the vascular layers of the eye, is one of the main areas of interest in utilizing BiTE therapy [74]. Many clinical trials are dedicated to developing new treatments due to its high hematogenous spread to the liver as well as its aggressive nature with limited treatment options [75]. It is estimated that 45% of patients diagnosed with uveal melanoma have the HLA-A*02:01 antigen [69]. Tebentafusp, a currently 2022 FDA-approved BiTE therapy, is a bispecific gp100 peptide that is used in HLA-A*02:01-positive adult patients with unresectable or metastatic uveal melanoma [76]. Unlike other traditional BiTEs, tebentafusp binds gp100 peptide-HLA complex instead of TAAs, thus increasing specificity towards tumor cells and decreasing overall side effects [77]. Tebentafusp recruits and activates polyclonal T-cells (via CD3) to release inflammatory cytokines and cytotoxic proteins, resulting in direct lysis of uveal melanoma tumor cells [78].

There are currently many clinical trials evaluating BiTE therapy in uveal melanoma. In a currently active phase II clinical trial, tebentafusp is evaluated against an investigator’s choice of therapy (Systemic Dacarbazine, Ipilimumab, or Pembrolizumab) in HLA-A*0201 positive patients with uveal melanoma with no prior systemic or liver-directed chemo-, radio- or immune-therapy administration (NCT03070392). The trial recently presented its three-year study analysis highlighting the efficacy and safety profile of tebentafusp against standard therapy. The OS in patients who received tebentafusp was 21.7 months compared to the investigator’s choice of 16 months with an HR of 0.51 (*p* < 0.001). At the three-year mark, the percentage of patients who survived in the tebentafusp group was 27%, whereas 18% survived in the control group. In the tebentafusp group, the most common TRAEs were rash (83%), pyrexia (76%), pruritus (70%), and hypotension (38%), with most TRAEs occurring early in the therapeutic course. Discontinuation due to TRAEs occurred in 2% of the patients in the tebentafusp group and 5% in the control group [79]. These results are imperative in demonstrating a long-term sustained clinical response as well as a safe profile in utilizing tebentafusp in uveal melanoma. Additionally, these data are especially important given the limited treatment options available for uveal melanoma patients as ICI has proven to be significantly less effective in this patient cohort [80].

Another multi-center phase I/II clinical trial recently completed its evaluation of tebentafusp in advanced uveal melanoma (NCT02570308). The trial demonstrated an ORR based on RECIST v1.1 criteria of 5%, a 1-year OS rate of 62%, and a median OS of 16.8 months. Therefore, based on these results, clinical benefit beyond radiographic response criteria could be evidenced through the OS rate. The study reported that every patient in the study experienced at least one TRAE with the most common TRAEs being rash (87%), pyrexia (80%), and pruritus (67%) [81]. Thus, this study highlights the viability of tebentafusp as a therapeutic option for advanced uveal melanoma based on the OS rate. Interestingly, the study found that early on-treatment reduction in circulating tumor DNA had strongly correlated with OS [81]. Therefore, these results demonstrate the need to further investigate the viability of circulating tumor DNA as a biomarker for tebentafusp response and highlight the need for further expansion of these results in phase III clinical trials.

Although they have demonstrated clinical efficacy in phase II trials, BiTE therapy presents with many challenges that limit its clinical efficacy in larger patient cohorts. Similar to other cell-based therapies, drug delivery remains a substantial challenge due to BiTE therapy’s short serum half-life. Thus, in order to have efficacious treatment, a continuous intravenous infusion is required [74,82]. To alleviate this challenge, techniques in optimizing drug delivery and altering pharmacokinetics BiTEs are currently being investigated. For instance, half-life-extended (HLE) BiTEs, which are BiTEs that comprise single-chain polypeptides with an added Fc region, forming a bispecific antibody with a higher molecular weight and longer half-life, have been explored in addressing this limitation. Similarly, studies on alternative administration routes, such as subcutaneous BiTEs, have shown a similar safety profile to the intravenous formulations and highlight another administration method to address the challenges present with the treatment administration [73,83].

Additionally, like other cell-based therapies, challenges with TAA specificity and therapeutic resistance have limited the clinical efficacy of BiTE therapy in advanced melanoma. As mentioned previously, many of the tumor antigens in solid tumors are also co-present in normal tissue, which can lead to on-target off-tumor toxicity [73,83]. Achieving a balance between maximizing therapeutic benefits and minimizing treatment-related toxicities remains an area that requires further investigation [84]. In addition, therapeutic resistance mechanisms against BiTE therapy have been shown which include upregulation of inhibitory immune checkpoints in the tumor microenvironment and downregulation or loss of TAA expression after starting the treatment [73,85,86]. Therefore, research is currently being undertaken to evaluate constructs that target the PD-1/PD-L1 axis to offset the upregulation of immune checkpoint inhibition as well as explore the potential synergism that may exist in combining BiTEs with ICIs [73,87].

Nevertheless, ongoing preclinical investigations and early-phase clinical trials in uveal melanoma treatment are progressing, indicating advancement in treatment options as many new clinical trials unfold (Table 3). This includes a phase III clinical trial that evaluates adjuvant tebentafusp in high-risk ocular melanoma (NCT06246149) and another phase III clinical trial that investigates the efficacy of tebentafusp in the neoadjuvant setting in uveal melanoma (NCT06414590). The expansion of phase III clinical trials that investigate tebentafusp in different clinical settings is especially important to help gain a picture of where tebentafusp could fit in the treatment strategy against uveal melanoma. More importantly, phase II/III clinical trials are especially needed to evaluate whether tebentafusp could demonstrate similar clinical efficacy in other skin malignancies such as cutaneous melanomas that are less responsive to standard therapies.

Additionally, another bispecific protein target that has gained research attention in melanoma is IMC-F106C. IMC-F106C is a TCR-transduced T-cell therapy that consists of both a TCR that specifically targets HLA-A*02:01 and preferentially expressed antigen in melanoma (PRAME), as well as an anti-CD3 scFv portion to attach to T-cells [88]. The PRAME protein is commonly found in several solid tumor subtypes, ranging from melanoma to breast cancer and lung cancer [89]. However, it is worth noting that PRAME is very highly expressed in melanoma, with over 95% expression in cutaneous melanoma and over 90% in uveal melanoma [90]. Therefore, the expression profile of PRAME in melanoma and the specificity of this new immunotherapy suggest its potential utility. The PRISM-MEL-301 phase III clinical trial is currently investigating the use of IMC-F106C in combination with nivolumab vs. nivolumab alone or nivolumab with relatlimab in the treatment of previously untreated advanced melanoma (NCT06112314). Future results from this large-scale clinical trial are highly anticipated, along with currently active Phase I trials (NCT05973487, NCT03686124) seeking to better characterize the utility of PRAME in the treatment of melanoma.

In conclusion, BiTE therapy represents a promising frontier in the treatment of melanoma, providing a targeted approach to activate T-cells against tumor cells. Earlier studies have focused on establishing safety profiles and maximum dosage, laying the foundation for ongoing and future trials. While challenges such as treatment-related toxicities and short half-life continue to persist, current clinical trials are paving the way for advancements. By exploring combination therapies and innovative strategies to enhance efficacy and safety and overcome resistance mechanisms, BiTE therapy holds the substantial potential to improve clinical outcomes and redefine treatment standards for patients with melanoma.

## 5. Conclusions

Melanoma treatment has greatly expanded in recent years due to the introduction of ICI and BRAF/MAPK-targeted therapies. However, many patients do not respond well to these standard therapeutics. Therefore, a call remains for therapeutic options beyond standard immunotherapy that address this gap in treatment options. 

The landscape of melanoma treatment has significantly advanced following the FDA approval of lifileucel. The T-cell-based therapy demonstrated significant efficacy in patients who failed standard ICI according to the C-144-01 study (NCT02360579), providing hope for the unmet need in patients who otherwise progress on standard therapies. Additionally, the treatment also demonstrated significantly higher efficacy against ipilimumab, the standard second-line treatment option in melanoma (NCT02278887), which sheds light on how lifileucel could fit in the treatment algorithm against melanoma. However, the treatment presents many challenges such as a long waiting time, high cost of production, and centralization in locations of expertise, and many patients are therefore precluded from benefiting from this therapeutic option. Additionally, no established biomarkers exist to address how to optimize this therapeutic approach in melanoma treatment. Therefore, more research is warranted to address the challenges presented by this therapeutic approach. 

Similarly, many other cell-based therapies are currently being evaluated for their efficacy and safety in advanced melanoma. In uveal melanoma, BiTE therapy has greatly widened the therapeutic options available for this distinct patient cohort who otherwise demonstrated limited responsiveness to standard therapies [91]. Indeed, BiTE therapy has demonstrated both sustained long-term responsiveness (NCT02570308) and significantly more clinical efficacy compared to standard second-line therapies (NCT03070392) in uveal melanoma. These promising phase II clinical trial results have paved the way for phase III trials to expand these findings in larger melanoma cohorts. However, the treatment comes with many challenges, including drug toxicities similar to other antigen-based therapies, optimal drug delivery, and therapeutic resistance, which require further research to better understand how to optimize this treatment approach in uveal melanoma. Nonetheless, BiTE therapy has evolved the treatment landscape of uveal melanoma and many research endeavors are being undertaken to evaluate how to optimize the treatment in melanoma therapy.

Importantly, there remains a need to explore the combination of cell-based therapies with other standard treatment options in advanced melanoma. Specifically, radiotherapy has demonstrated great efficacy in being combined with systemic therapies due to its immunostimulatory effect [92]. Radiotherapy has been implicated in upregulating MHC-1 expression on tumor surfaces and therefore increasing tumor antigen presentation. Furthermore, radiation therapy can initiate the priming of T-cells against tumor cells [93]. These immunostimulatory effects provide convincing evidence for the need for exploring the combination of cell-based therapies and radiotherapy. This combinatory approach could further extrapolate the efficacy of cell-based therapies through post-escape radiotherapy, where the combination of radiotherapy and cell-based therapies are administered following tumor escape of the immune system [92]. However, a greater understanding of the biological mechanisms underlying this combinatory approach is still needed to identify patients who may benefit from this approach as well as to explore potential synergism that may exist between these therapeutic options. Thus, convincing evidence exists in combining cell-based therapies with radiotherapy; however, more research is warranted to explore this approach in preclinical and clinical studies.

In conclusion, cell-based therapies have shown promise in expanding the treatment options available for patients who otherwise do not benefit from standard immunotherapy. The recent FDA approval of lifileucel has sparked great interest in understanding how this new treatment could fit into the fight against melanoma. Yet, much research is needed to address the challenges present with cell-based therapies to better augment their clinical efficacy in melanoma and other solid tumors.

## Figures and Tables

**Table 1:** TIL therapy ongoing clinical trials in melanoma.

Study Name (NCT Number)	Trial Stage	Therapeutic Approach	Enrollment	Disease/Stage
ACTME (NCT04330430)	Phase I/II (Active, Not Recruiting)	One cohort was given a combination of TILs and anti-PD-1 and the second was given TILs and anti-PD-1 with PEG-IFNa	34	Unresectable stage III–IV melanoma
TIL (NCT02278887)	Phase III (Active, Not Recruiting)	TIL treatment vs Ipilimumab	168	Unresectable stage III–IV melanoma
(NCT05176470)	Phase I (Active, Not Recruiting)	Neoadjuvant administration of TIL therapy with Pembrolizumab	2	Stage IIIB-D to stage IV melanoma
(NCT01005745)	N/A (Active, Not Recruiting)	TILs with high dose IL-2	19	Unresectable metastatic stage IV melanoma or stage III in-transit or regional nodal disease
LN-144 (NCT02360579)	Phase II (Active, Not Recruiting)	Lifileucel	178	Unresectable or metastatic (Stage IIIc or IV) melanoma
(NCT05470283)	Phase I (Active, Not Recruiting)	TILs engineered with membrane-bound IL15 plus acetazolamide	21	Unresectable stage III–IV melanoma
mMEL (NCT03475134)	Phase I (Active, Not Recruiting)	TILs followed with or without nivolumab rescue	18	Unresectable stage IIIc–IV melanoma who have progressed on at least 1 standard first-line therapy
TUNINTIL (NCT04217473)	Phase I (Active, Not Recruiting)	TNFalpha and IL-2 coding oncolytic adenovirus TILT-123 and TILs therapy	17	Previously treated refractory or recurrent stage 3–4 melanoma
(NCT01659151)	Phase II (Active, Not Recruiting)	Vemurafenib with TILs therapy and high dose IL-2	17	Unresectable metastatic stage IV melanoma or stage III in-transit or regional nodal disease
(NCT00338377)	Phase II (Active, Not Recruiting)	TIL therapy with or without dendritic cell immunization	1230	Metastatic melanoma, uveal melanoma, or stage III in-transit or regional nodal disease
(NCT01319565)	Phase II (Active, Not Recruiting)	TILs plus IL-2 following either a non-myeloablative Lymphocyte Depleting chemotherapy regimen alone or in combination with 12Gy total body irradiation	102	Metastatic melanoma with at least one lesion resectable for TIL generation
(NCT01701674)	N/A	TILs plus IL-2 with Ipilimumab	13	Unresectable metastatic stage IV melanoma or stage III in-transit or regional nodal disease

Abbreviations: N/A = not available.

**Table 2:** Ongoing clinical trials in melanoma for modified cell therapies.

Study Name (NCT Number)	Trial Stage	Therapeutic Approach	Enrollment	Disease/Stage
(NCT02870244)	Phase I (Recruiting)	TIL 1383I TCR transduced T-cells	18	Advanced melanoma
(NCT01586403)	Phase I (Active, not recruiting)	Two doses of autologous T-cell receptor transduced T-cells, administered along with IL-2	14	Stage IV melanoma patients
(NCT02650986)	Phase I/II (Active, not recruiting)	Genetically modified T-cells, with or without concurrent decitabine	15	Advanced melanoma, ovarian, fallopian, peritoneal, and synovial cancers
(NCT02869217)	Phase I (Active, not recruiting)	TBI-1301 (TCR gene transduced autologous T lymphocyte) after cyclophosphamide and fludarabine pre-treatment	22	Solid tumors, including melanoma
(NCT05296564)	Phase I/II (Recruiting)	HBI-0201 (TCR gene-engineered lymphocytes)	3	NY-ESO-1 expressing metastatic cancers, including stage IV melanoma
(NCT03132922)	Phase I (Active, not recruiting)	MAGE-A4c1032 modified T-cell therapy	71	HLA-A2 positive participants with MAGE-A4 positive tumors, including melanoma
(NCT04897321)	Phase I (Recruiting)	B7-H3-specific CAR T-cells, administered after lymphodepleting chemotherapy	32	B7-H3-positive tumors, including melanoma
(NCT04483778)	Phase I (Active, not recruiting)	B7H3-specific CAR T-cells vs. B7H3-CD19-specific CAR T-cells vs. B7H3-CD19-specific CAR T-cells and pembrolizumab	68	Recurrent/refractory solid tumors, including melanoma in children and young adults
(NCT04119024)	Phase I (Recruiting)	IL13Ralpha2 CAR T-cells	18	Stage IIIc or IV melanoma and other metastatic solid tumors
(NCT04729543)	Phase I/II (Recruiting)	MAGE-C2/HLA-A2 TCR T-cells	20	Melanoma and head and neck cancer
(NCT05415072)	Phase I/II (Recruiting)	Single-agent DYP688	124	Metastatic uveal melanoma (MUM) and other melanomas with GNAQ/11 mutations
(NCT05588453)	Phase I/II (Recruiting)	Natural killer cell therapy (UD TGFbetai NK cells) and temozolomide	30	Stage IV melanoma metastatic to the brain
(NCT05629546)	Phase I (Not Yet Recruiting)	Autologous: Memory-like natural killer cells + nivolumab + relatilimab and Allogeneic: Memory-like natural killer cells + nivolumab + relatilimab	33	Advanced or metastatic melanoma that has progressed after at least 12 weeks or a minimum of 2 doses of treatment with a standard of care
(NCT03420963)	Phase I (Recruiting)	Cyclophosphamide + etoposide + NK cells	38	Patients with relapsed or refractory solid tumors

**Table 3:** Ongoing BiTE therapy clinical trials in melanoma.

Study Name (NCT Number)	Trial Stage	Therapeutic Approach	Enrollment	Disease/Stage
(NCT05315258)	Phase II (Recruiting)	Tebentafusp	850	Cutaneous melanoma with molecular relapsed disease (MRD) and uveal melanoma with MRD
(NCT05549297)	Phase II/III (Recruiting)	Tebentafusp as a monotherapy or in combination with pembrolizumab vs investigator’s choice	460	HLA-A*02:01-positive with previously treated non-ocular advanced melanoma
TEBE-AM (NCT05549297)	Phase II/III (Recruiting)	Tebentafusp monotherapy in combination with pembrolizumab vs. standard of care	460	Previously treated advanced melanoma
(NCT06070012)	Phase II (Not yet recruiting)	Tebentafusp	44	Previously untreated metastatic uveal melanoma with an integrated circulating tumor DNA biomarker
(NCT06414590)	Phase II (Not yet recruiting)	Neoadjuvant tebentafusp	19	Locally Advanced, Unresectable Primary Uveal Melanoma
(NCT03070392)	Phase II (Active, not recruiting)	Tebentafusp vs investigator’s choice (Dacarbazine, ipilimumab, or pembrolizumab)	378	Previously untreated advanced uveal melanoma
(NCT02570308)	Phase I/II (Completed)	Tebentafusp	146	Advanced uveal melanoma with (HLA)-A*0201 positive
PRISM-MEL-301 (NCT06112314)	Phase III (Recruiting)	IMC-F106C plus nivolumab vs. nivolumab and relatlimab vs. nivolumab alone	680	Previously untreated advanced melanoma
ACTengine (NCT03686124)	Phase I (Recruiting)	IMA203/IMA203CD8 products, with or without combination nivolumab	186	Solid tumors that express PRAME
(NCT05973487)	Phase I (Recruiting)	T-Plex, an autologous customized TCR therapy targeting specific peptide/HLA antigens	100	Locally advanced (unresectable) or metastatic solid tumors

## Data Availability

No new data were created or analyzed in this study.
